# A Working Program for the Extinction of the Defective Delinquent

**Published:** 1913-07

**Authors:** Hastings H. Hart

**Affiliations:** Director of the Department of Child Helping of the Russell Sage Foundation


### A Working Program for the Extinction of the Defective Delinquent.

BY HASTINGS H. HART,

DIRECTOR OF THE DEPARTMENT OF CHILD HELPING OF THE RUSSELL SAGE FOUNDATION.

In every prison and reformatory are found insane, feeble-minded, epileptic, alcoholic, "drug fiends" or cripples. The term defective delinquents is applied to such individuals. Until recently there was no reliable information as to the number of defectives in prisons and reformatories.

Scientific psychological studies of the inmates of several adult and juvenile reformatories indicate that the number of the feeble-minded in reformatories is much larger than had been supposed. Dr. Walter E. Fernald of the Massachusetts School for the Feeble-minded says: "At least 25 per cent of the inmates of our penal institutions are mentally defective. Nearly 50 per cent of the girls at the Lancaster Reformatory are mentally defective."

Psychological examinations of inmates of a number of reformatories indicate defectives, approximately, as follows:

New York Reformatory, Elmira, 37 per cent.
New York Reformatory, Rahway, 33 per cent.
New York Reformatory for Women, Bedford, 37 per cent.
Massachusetts Industrial School for Girls, Lancaster, 50 per cent.
Maryland Industrial School for Girls, Baltimore, 60 per cent.
State Home for Girls, Trenton, 33 per cent.
Illinois State School for Boys, St. Charles, 20 per cent.

Judging from these figures, Dr. Fernald's estimate of 25 per cent is conservative. On that basis there are 20,000 defective delinquents in adult prisons and 6,000 in juvenile reformatories, or a total of 26,000 in actual custody. Probably as many more are at large as there are in institutions.

Thus we have two classes of inmates: people able to take care of themselves if a proper basis of character could be established; and people so deficient in mentality as to be unable to succeed.

These two classes need radically different treatment. The normal inmate needs physical renovation, intellectual stimulus, educational treatment, religious training, proper desires, self-control and self-reliance, vocational training, release on parole, restoration to family life. But the defectives in prisons and reformatories need kindly care, rudimentary education, physical training, vocational training in simple industries, plenty of recreation and sympathetic care, and permanent institutional life. It is a needless cruelty to discipline, exhort and educate the feeble-minded in fruitless efforts to develop faculties of mind and soul which do not exist.

In order to extinguish defective delinquents we must restrict the propagation of the feeble-minded. This may be undertaken by popular education in the principles of eugenics, by laws restricting marriage, by the sterilization of defectives and by segregation.

Education against the marriage of the unfit reaches only the intelligent, not the most dangerous. Restrictive marriage laws are unavailing because the unfit reproduce their kind regardless of marriage laws. Sterilization is at best a partial remedy, and is restricted in application by public sentiment. It is actually operative in only one of the eight States which have passed sterilization laws.

STERILIZATION.

Sterilization is advocated by many penologists and alienists as a preventive measure. The writer, it should be stated, in order to avoid misunderstanding, is one of those who favors the sterilization of rapists, sexual perverts or degenerates, confirmed masturbators and others whose sexual tendencies call for such action for the protection of the community.

Laws providing for the sterilization of certain classes have been passed by eight States: Indiana (1907); Connecticut, California and Washington (1909); Nevada, New Jersey and Iowa (1911); and New York (1912). Most of these acts have followed the general trend of the Indiana law, which provides that each institution entrusted with the care of confirmed criminals, idiots, rapists and imbeciles must appoint two skilled surgeons. These, with the chief physician of the institution, are directed to examine the mental and physical condition of such inmates as are recommended by the institutional physician and board of managers. If, in the judgment of this committee and the board of managers, procreation is inadvisable and there is no probability of improvement it shall be lawful to perform such operation for the prevention of procreation as shall be decided safest and most effective.

The New Jersey law covers rapists, idiots, imbeciles, morons, epileptics and other defectives.

The provision of the laws passed in other States are similar to those of Indiana and New Jersey. The Connecticut and the Iowa laws impose a penalty upon a surgeon who performs such an operation upon any person outside the classes described in the act.

The Iowa law adds drunkards, drug fiends and syphilitics.

The objects of sterilization laws are stated as follows:

(a) To prevent criminal heredity.

(b) To prevent rape and punish rapists.

(c) To prevent the inheritance of feeble-mindedness, epilepsy, etc.

(d) To provide "a healthful warning and deterrent for reckless sex defectives."

Dr. E. C. Sharp, physician of the Indiana State Reformatory at Jeffersonville, in a pamphlet published by the National Christian League for the Promotion of Purity, considers "means by which we may beget none but sound offspring."

He discusses proposed methods and says: "Restricting propagation seems to be universally agreed upon as necessary for the relief of this condition. The difficulty lies in deciding upon proper method." He discards several proposed methods, as follows:

(a) "The education of public opinion, so that those who are from defective parentage shall abstain from marriage" because it "is even worse than absurd."

(b) Restrictive legislation for the purpose of preventing marriage among defectives because, unfortunately, "matrimony is not always necessary to propagation."

(c) Segregation of degenerates in institutions because it would necessitate the expenditure of enormous sums to maintain colonies or industrial refuges, which would be a disappointment in the end.

(d) "Castration" because "it causes too much mental and nervous disturbance."

Dr. Sharp says: "I heartily endorse as an additional punishment in certain offenses an operation known as vasectony, which consists of ligating and resecting a small portion of the vas deferens."

(a) "This operation is simple and easy without anesthetic, general or local."

(b) "The subject is effectually sterilized."

(c) "He returns to work immediately, suffers no inconvenience, and is in no way impaired for his pursuit of life, liberty and happiness."

(d) "There is no disturbed mental or nervous condition following."

(e) "The patient ceases excessive masturbation."

(f) "He advises his fellows to submit to the operation for their own good."

(g) "After the vas deferens has been severed you may, by a second operation, repair it and re-establish the original function."

(h) "Women may be subject to sterilization as well as men."

It is astonishing that this plan should have been adopted by so many legislatures in view of the fact that it has been discredited in Dr. Sharp's own State, and has been put into operation thus far in only one other State.

The Indiana law went into effect in 1907. The published reports of the Indiana State Reformatory show in the fiscal year 1907-8, 119 operations; in 1908-9, thirty-nine operations; in 1909-10, one operation; in 1910-11, none. The reason for the discontinuance of this operation, notwithstanding the fact that the law said "it shall be compulsory for each and every institution," is that the State of Indiana believed the law unconstitutional. Up to November 1. 1912, there has been no operations under the laws of Connecticut, Washington, New Jersey, Iowa or New York. In California the law has gone into effect, and more than 300 operations have been performed.

In view of these facts, it would seem wise for other States to await judicial decisions and practical tests of this plan in States where the law has already been adopted before enacting such legislation. Certain objections to the method proposed by Dr. Sharp present themselves to the practical student:

(1) The operation has little, if any, deterrent value. Dr. Sharp says: "All other methods proposed place restrictions—therefore punishment—upon the subject; this method absolutely does not." In another article he calls attention to the fact that the disability can be removed by a second operation.

(2) This operation, while it sterilizes the individual, does not interfere with the practice of sexual intercourse; it does not prevent the individual from committing rape; it does not prevent masturbation. (Dr. Sharp says: "The patient . . . ceases excessive masturbation.") It does not interfere in the slightest degree with the transmission of venereal disease.

(3) The plan advocated by Dr. Sharp carries with it a proposition which is monstrous and abhorrent. After setting forth the contents and the effect of the Indiana law, he says: "This is indeed a very long step in the right direction. . . . I would, however, carry it a little further, and make the provision in our marriage laws that when one or both contracting parties suffer from a defect, or a chronic transmissible disease, the male should be sterilized. Then let them go on and marry; and by this means there will possibly be a support given and a protectorate thrown about some feeble-minded woman, that in any other event would become a public charge, or a prostitute, or more likely the mother of illegitimate children."

Consider the meaning of this last declaration. The feeble-minded girl is, in physical development and inclinations, a woman; she is in mind an innocent child. Dr. Sharp proposes to take this innocent and helpless child and hand her over bodily to a diseased rake, "and by this means there will possibly be a support given and a protectorate thrown about some feeble-minded woman." It is time that a protest was raised against this inhuman proposition.

Sterilization is a legitimate and proper proceeding for certain classes; but the method employed should be such that it will be dreaded and not welcomed by the criminal and the degenerate, and such as to hinder and not to promote the spread of disease. No sterilization law can be effective until a strong public sentiment is developed to sustain the execution of such laws.

### SEGREGATION—THE METHOD CITED.

Segregation is the most practical and effective means to restrict the propagation of the feeble-minded. It has been successfully tested with the insane. Thirty years ago the segregation of the insane seemed almost a hopeless undertaking, but, in twenty-three years, from 1880 to 1903, the number of insane hospitals was increased nearly four-fold, and the ratio was increased from eighty-two to 186 for each 100,000 of the population. What has been done for the insane can be done for the feeble-minded.

We may estimate the number of feeble-minded under public care as follows: In institutions for feeble-minded, 20,000; in almshouses, 16,000; in hospitals for insane, 5,000; in prisons and reformatories, 26,000. Thus 67,000 are already under public care or as near as can be judged one-third of the feeble-minded persons in the United States. The problem of segregating the feeble-minded is not as large in proportion to our resources as was that of segregating the insane thirty years ago.

### A PRACTICAL WORKING PROGRAM.

Thus far we have started at the wrong end. Schools have been built on the theory that, by employing teachers of special skill and training and by adopting improved educational methods, defective children will become normal members of the community.

These hopes have invariably been disappointed. When these children have been sent out into the world, they have either returned or have become a burden upon the community in other ways. The most competent authorities agree that it is useless to try to develop latent mentality of feeble-minded children, because it does not exist.

The following suggestions are offered as a working program:

(1) Secure legislation whereby institutions for feeble-minded children shall hold their inmates as the insane are, by legal commitment. In most States, the parents' consent to commitment is either required or is customary. Parents who find their children apparently improved often insist upon their release against professional advice.

(2) Secure legislation whereby whenever inmates of institutions for other classes are found to be feeble-minded, they may be kept permanently in public care.

(3) Provide by law for the establishment of separate departments, in connection with prisons and reformatories, and transfer to these colonies for permanent custodial care all inmates found to be feeble-minded.

(4) Convert existing institutions which are no longer needed for present purposes into State institutions for defective delinquents.

(5) Undertake a comprehensive campaign for the care of all feeble-minded girls of child-bearing age. The problem of the feeble-minded girl is much more acute than that of the feeble-minded boy. In a letter just received from Dr. Henry H. Goddard, of New Jersey, he says: "The feeble-minded woman is more dangerous to society than the feeble-minded man, because she is much more likely to find a mate than he is—possibly, according to our statistics, somewhere in the neighborhood of three times as likely." There are probably 60,000 feeble-minded women of child-bearing age in the United States. About 13,000 are already under care of reformatories and institutions for the feeble-minded. We are already taking care of about 100,000 insane women in hospitals. It is possible to make institutional provision for all the feeble-minded women in the United States.

(6) Secure legislation whereby institutions for feeble-minded shall cease to receive girls under the age of twelve, or boys of any age, until every feeble-minded girl of child-bearing age is provided for. The feeble-minded girl is but a child. She is as innocent, helpless and child-like as her normal sister of half her years, and she is entitled to the same protection and chivalrous regard as the little girl of similar mentality. Yet she is left without protection to be pursued and hunted by evil-minded men as ruthlessly as hunters pursue a rabbit. Society deals with her as if she were responsible. She is arrested, condemned to prison, or, more humanely, sent to a reformatory for discipline and training; but the managers are compelled by law to send her home to be exposed to fresh temptation, abuse and contumely, to breed her own sort, and finally to join the great army of prostitutes.

(7) Undertake a vigorous campaign throughout the country for increased provision for the feeble-minded classes. This is the next great task of our people, and it must be bravely met.

This campaign should undertake to establish:

(a) Care in almshouses for males over fourteen years and females over forty-five.

(b) Custodial institutions for feeble-minded women like those in New York and New Jersey.

(c) Institutions for defective delinquents among those committed by the criminal and juvenile courts.

(d) Small institutions for young children who ought to be cared for as a matter of humanity.

(e) Schools for backward children in the large cities to sift out subnormal children, and give them schoolroom care until institutional care can be provided. In small communities it is impossible to establish such schools, and the only practical way to meet their needs is by creating State or county institutions.—*The Survey*, May 24th.

